# One thousand patients with essential thrombocythemia: the Florence-CRIMM experience

**DOI:** 10.1038/s41408-023-00968-7

**Published:** 2024-01-18

**Authors:** Giuseppe G. Loscocco, Francesca Gesullo, Giulio Capecchi, Alessandro Atanasio, Chiara Maccari, Francesco Mannelli, Alessandro M. Vannucchi, Paola Guglielmelli

**Affiliations:** 1 Department of Experimental and Clinical Medicine, CRIMM, Center of Research and Innovation of Myeloproliferative Neoplasms, Azienda Ospedaliero-Universitaria Careggi, University of Florence, Florence, Italy; 2 Doctorate School GenOMec, University of Siena, Siena, Italy

**Keywords:** Risk factors, Myeloproliferative disease

## Abstract

We describe 1000 patients with essential thrombocythemia seen at the Center Research and Innovation of Myeloproliferative Neoplasms (CRIMM), Florence, Italy, between 1980 and 2023: median age 59 years (18–95), females 65%, *JAK2*/*CALR*/*MPL*-mutated 66%/19%/4%, triple-negative (TN) 11%. Extreme thrombocytosis (ExT, platelets ≥1000 × 10^9^/L) in 16%, leukocytosis (leukocytes >11 × 10^9^/L) in 16%, and at least one cardiovascular risk factor in 52% of cases. *JAK2*-mutated patients were older (median 62 years) and *CALR*-mutated and TN (53 years for both) younger (*p* < 0.001). Female gender clustered with TN (76%) and *JAK2* (67%) vs *CALR* (46%) mutations (*p* < 0.001). ExT clustered with *CALR* (type-2 more than type-1), TN and *MPL*, and leukocytosis with *JAK2* mutation (*p* < 0.001). In multivariable analysis, risk factors for arterial thrombosis-free survival were age ≥60 years (HR 2.0; *p* < 0.001) and *JAK2* mutation (HR 1.3; *p* = 0.02) with borderline significance for male gender (*p* = 0.08) and cardiovascular risk factors (*p* = 0.08); for venous thrombosis-free survival, *JAK2* mutation (HR 1.9; *p* = 0.03) with borderline significance for venous thrombosis history (*p* = 0.07); for overall survival, older age (*p* < 0.001), male gender (HR 1.9; *p* < 0.001), absolute neutrophil count (ANC) ≥ 8 × 10^9^/L (HR 1.8; *p* = 0.01), absolute lymphocyte count (ALC) < 1.7 × 10^9^/L (HR 1.2; *p* = 0.03); for myelofibrosis-free survival, *CALR* mutation (HR 2.7; *p* < 0.001, particularly for *CALR* type 1/1-like, HR 3.3) and *MPL* mutation (HR 3.9; *p* = 0.001); for leukemia-free survival, older age (*p* = 0.03). Cytoreductive therapy appeared to mitigate both venous (HR 0.3; *p* = 0.01) and arterial thrombosis (HR 4; *p* = 0.04); there was a trend for aspirin in preventing arterial thrombosis recurrence. The current study provides real-world observations in essential thrombocythemia, representing a valid source document for interpreting current literature and planning future studies.

## Introduction

Essential thrombocythemia (ET), first described by Epstein and Goedel in 1934 [1], is one of the classic Philadelphia-negative myeloproliferative neoplasms (MPNs), along with polycythemia vera (PV), prefibrotic and overtly fibrotic stage primary myelofibrosis (PMF), and MPN unclassifiable (MPN-U)/not otherwise specified (MPN-NOS) according to the latest International consensus (ICC) [2] and World Health Organization (WHO) [3] classification systems. Furthermore, ET may develop a fibrotic phenotype over time, referred to as post-ET myelofibrosis (MF) [2, 4].

ET is mainly characterized by the proliferation of abnormal megakaryocytes in the bone marrow (BM), resulting in an increased number of circulating platelets. Clinical manifestations include vasomotor symptoms such as erythromelalgia, migraine, and paresthesia, constitutional symptoms, and complications from thrombosis, both arterial and venous, and bleeding; mild (<5 cm from left costal margin) splenomegaly can be seen in about 15–20% of the cases, while hepatomegaly is rare [5].

Rates of complications are relatively high. Of note, the incidence of thrombotic complications ranges from 9% to 84% at diagnosis and from 7% to 32% during long-term follow-up in various settings and using different definitions of events. For bleeding, the respective rates range from 4% to 63% and from 8% to 14% [6–8]. However, approximately 40% to 50% of patients are asymptomatic at diagnosis, and thrombocytosis is an incidental finding on routine blood testing [9]. Overall, ET has the most favorable prognosis among MPN but presents considerable clinical heterogeneity, and a minority of patients who develop progression to MF or acute myeloid leukemia (AML), referred to as blast-phase (BP) MPN, have a much poorer outcome [9].

Diagnosis of ET is made on the basis of patterns of histopathology in the BM, which is typically normocellular for patients’ age and has an increased number of mostly mature-appearing megakaryocytes, either sparse and constituting loose clusters and reticulin fibrosis <grade 1; demonstration of clonality and/or the absence of a clear secondary cause of thrombocytosis, and exclusion of other hematologic disorders with thrombocytosis, are required diagnostic criteria [10]. Among conditions associated with secondary/reactive thrombocytosis, the most frequent are infections, iron deficiency, chronic inflammation, drugs and splenectomy [11].

The hallmark of ET clonality is represented by the presence of driver mutations, which are usually mutually exclusive and responsible for constitutive activation of the JAK-STAT signaling pathway. They include *JAK2* V617F, exon 9 calreticulin (*CALR*) comprising type 1/1-like, type 2/2-like, and thrombopoietin receptor (*MPL* W515L/K/A) mutations in approximately 55–65%, 20–25% and 5–8% of cases, respectively. Of note, a driver mutation is missing in 10–15% of ET patients, which are referred to as triple negative (TN) [12].

Current risk stratification in ET addresses the likelihood of thrombosis and relies on the international prognostic score in essential thrombocythemia (IPSET) for thrombosis, which includes age >60 years, thrombosis history, cardiovascular risk factors and *JAK2*V617F mutation, as risk variables [13]. The score was re-analyzed and led to a refined four-tiered, revised version, which excluded cardiovascular risk factors [14]; this score was validated in a large independent cohort [15]. On the other hand, prediction for survival in ET is conventionally based on clinical risk variables, including age, thrombosis history and leukocyte count [16]. Recent collaborative study between Mayo Clinic, Rochester, USA, and the University of Florence, Italy, led to an integrated clinical and genetic survival risk model for ET (MIPSS-ET), by identifying the presence of *SF3B1* and *SRSF2* mutations (occurring in ∼10% of patients), age >60 years, male sex, and leukocytosis (≥11 × 10^9^/L) as independent risk factors for reduced overall survival [17]; this collaborative effort also identified *SF3B1* and *U2AF1* mutations as risk factors for fibrotic progression and highlighted the protective role on arterial thrombosis of *ASXL1*, *RUNX1* and *EZH2* mutations [18]. Moreover, ET patients with *JAK2*V617F with a variant allele frequency (VAF) > 35%, *CALR* type 1/1‐like or *MPL* mutations were defined at high risk for MF progression [19]. More recently, the 4-tiered “triple AAA” risk model considering Absolute Neutrophil count (ANC), Absolute Lymphocyte count (ALC) as well as age as risk variables was developed; four adverse points were attributed for age >70 years, two points for age 50–70 years, and one point each for ALC < 1.7 × 10^9^/L and ANC ≥ 8 × 10^9^/L [20, 21]. This score, while performing better than the current standard IPSET model and being more globally applicable than the MIPSS-ET model, also suggests a potential role for immune-related biomarkers as a prognostic tool in ET [21].

The present paper summarizes the last four decades of our experience with patients with ET as a tertiary reference center. We considered 1000 consecutive ET patients who were seen from March 1980 to September 2023 and in whom clinical and bone marrow histopathology information was available for review, together with molecular characterization and long-term follow-up data. Our aims were to define (1) presenting clinical and laboratory features for patients seen at the time of diagnosis, (2) the natural history of the disease, including the incidence of arterial and venous thrombosis at the time of diagnosis and during follow-up, the overall, myelofibrosis-free and leukemia-free survival, all interpreted in the context of contemporary prognostic scoring systems variables. We believe this set of data might serve as a valuable epidemiological resource for patients and physicians, as well as provide context for the design and interpretation of prospective and retrospective trials.

## Patients and methods

This retrospective study was approved by the Institutional review board of the Local Ethics Committee at Azienda Ospedaliero-Universitaria Careggi (Florence, Italy; Mynerva project, #14560) and was conducted in accordance with the ethical guidelines of the Declaration of Helsinki. Informed consent was obtained per institutional requirements. The study population consisted of ET patients diagnosed and/or reviewed according to the 2022 ICC and 5th edition WHO classification of myeloid neoplasms [2, 3], who were routinely followed at the Center Research and Innovation of Myeloproliferative Neoplasms (CRIMM), Azienda Ospedaliera Universitaria Careggi and University of Florence, Italy. Accordingly, bone marrow biopsies at the time of diagnosis were reviewed in order to avoid inadvertent inclusion of patients with prefibrotic MF. All patients were followed until death or last follow-up, as assessed by medical records or through direct contact with patients or their physicians. All patients were annotated for driver mutations comprising *JAK2*V617F, *CALR* exon 9 and *MPL* W515 in DNA in peripheral blood (PB) granulocytes as previously described [22]. Cytogenetic analysis and reporting were according to the International System for Human Cytogenetic Nomenclature criteria using standardized techniques [23].

Major Arterial thrombosis includes myocardial infarction, angina, cerebrovascular accidents, transient ischemic attack, peripheral arterial thrombosis, aortic thrombosis, mesenteric artery thrombosis, whereas major venous thrombosis includes deep venous thrombosis, pulmonary embolism, portal/splenic/mesenteric/hepatic vein thrombosis, cerebral sinus thrombosis, central retinal vein thrombosis. Thrombotic events were considered post diagnosis if they occurred at least 4 weeks after ET diagnosis, whereas thrombotic events before diagnosis included all events that occurred within 2 years prior to diagnosis. Microcirculatory symptoms included dizziness, headaches, visual disturbances, erythromelalgia, distal paresthesia and acrocyanosis. Major bleedings were defined based on the International Society on Thrombosis and Hemostasis (ISTH) definition as: gastrointestinal, internal organ, intraarticular, cerebrovascular, retroperitoneal bleed or any bleeding requiring medical and/or surgical intervention, hospitalization and/or resulting in death [24].

Statistical analyses included clinical and laboratory parameters obtained at diagnosis or first referral, in any case less than 1 year from diagnosis and without treatment apart from aspirin or anticoagulation. Continuous variables were presented as the median (range) and categorical variables as the frequency (percentage). Differences in the distribution of continuous variables in the categories were compared using the Mann-Whitney *U* test. The χ2 test was used for comparison of categorical variables. The Cox proportional hazard regression model was used for univariate and multivariate analysis and to generate hazard ratios and 95% confidence intervals (CIs). Overall survival analysis was considered from the date of diagnosis to the date of death or last contact. Arterial and venous thrombosis-free, myelofibrosis-free and leukemia-free survival calculations considered the event as the uncensored variable. The Kaplan–Meier method was used to construct time-to-event curves, which were compared using a log-rank test. Statistical analyses were performed with SPSS software, version 27 (IBM-Corp), JMP Pro 15.1.0 software from SAS Institute (Cary, NC) and Statistical Package R version 4.1.1.

## Results

### Patients’ characteristics at diagnosis

The study core was constituted of 1000 consecutive ET patients fully annotated for driver mutations; clinical information at disease presentation concerning blood counts, presence of cardiovascular risk factors including diabetes, hypertension, smoking and hyperlipidemia, palpable splenomegaly, microvascular symptoms, major thromboses and hemorrhages were available for all the patients. Median age at diagnosis was 59 years (range 18–95), and 646 (65%) were female. Median hemoglobin, leukocytes and platelet count values were 14 g/dL, 8.5 × 10^9^/L and 715 × 10^9^/L, respectively. 160 patients (16%) had a leukocyte count >11 × 10^9^/L, whereas 156 (16%) and 96 (10%) had a platelet count ≥1000 × 10^9^/L and ≥1500 × 10^9^/L, respectively. ANC and ALC values were available for 514 patients; correspondent median (range) values were 5.15 (1.7–19.2) (ANC ≥ 8 × 10^9^/L in 50 patients, 10%) and 2.0 (0.1–5.82) (ALC < 1.7 × 10^9^/L in 182 patients, 35%). A total of 126 patients (13%) had a palpable splenomegaly and 293 (29%) presented microvascular symptoms. The presence of at least one cardiovascular risk factor was reported in 518 patients (52%); the most common was hypertension (35%), followed by hyperlipidemia (18%), smoking (16%) and diabetes (6%). A driver mutation was found in 89% of patients: *JAK2* V617F in 659 patients (66%), exon 9 *CALR* in 187 patients (19%) comprising 115 *CALR* type 1/1-like and 72 *CALR* type 2/2-like, and *MPL* W515x in 44 cases (4%); accordingly, 11% were TN. Cytogenetic information was available in 299 cases (30%), and an abnormal karyotype was reported in 30 patients (10%).

Overall, major thromboses at or prior to diagnosis were documented for 186 patients (19%), including arterial (*n* = 132; 13%) and venous (*n* = 63; 6%) events. Major hemorrhages were reported in 42 (4%) patients. Moreover, class distribution according to the original IPSET thrombosis model was as follows: low (*n* = 244, 25%), intermediate (*n* = 221; 22%) and high (*n* = 535; 54%), whereas the revised version of IPSET thrombosis ranked 200 patients as very low (20%), 248 low (25%), 104 intermediate (10%) and 448 high (45%). Concerning treatment, antiplatelet therapy, which consisted of 100 mg/daily aspirin for most patients, was initiated at the time of diagnosis in 897 (90%), cytoreductive therapy (hydroxyurea in most instances) in 701 (70%), and systemic anticoagulation in 111 (11%). As regarded overall survival, risk class distribution according to the International Prognostic Scoring System for survival in ET (IPSET) was low in 38% of patients, intermediate in 44%, and high in 18%.

At presentation, *JAK2* mutated patients were older (median age 62 years), compared to *CALR* (median age 53 years; *p* < 0.001) and TN (median age 53 years; *p* < 0.001). In addition, *CALR* mutated patients were mostly male (*n* = 100/187; 54%) compared to 24% in TN (26/110; *p* < 0.001) and 33% in *JAK2* mutated cases (219/659; *p* < 0.001). Moreover, *JAK2* mutated patients displayed significantly higher hemoglobin levels (14.2 g/dL vs 13.8 g/dL; *p* < 0.001), higher leukocyte counts (8.7 × 10^9^/L vs 8 × 10^9^/L; *p* < 0.001) and lower platelet counts (671 × 10^9^/L vs 826 × 10^9^/L; *p* < 0.001) compared to *CALR* patients. Similar to *CALR* mutated, TN patients had lower hemoglobin levels (13.8 g/dL; *p* < 0.001) and higher platelet counts (725 × 10^9^/L; *p* = 0.005) compared to *JAK2* mutated cases. The number of major thromboses at or prior to diagnosis was significantly higher in *JAK2* patients (*n* = 149, 23%) compared to *CALR* (*n* = 16, 9%; *p* < 0.001) and TN (*n* = 11, 10%; *p* = 0.003). These differences between *JAK2* and *CALR* mutated remained significant when considering arterial (*p* = 0.001) and venous events (*p* = 0.007) separately. On the other hand, concerning major hemorrhages at or before ET diagnosis, there was no significant difference.

Compared to *JAK2* mutated cases, *CALR* type 1/1-like and *CALR* type 2-2-like mutated patients were younger (*p* = 0.03; *p* < 0.001) and displayed lower hemoglobin levels (*p* < 0.001; *p* < 0.001), lower leukocyte counts (*p* = 0.002; *p* < 0.001), and higher platelet counts (*p* < 0.001; *p* < 0.001). Notably, compared to the *JAK2* mutation, *CALR* type 1/1-like (*p* < 0.001) and type 2/2-like (*p* = 0.03) mutations were both associated with male gender. When type 1/1-like and type 2/2-like *CALR* mutations were directly compared to each other, the latter variants displayed significantly higher platelet counts (*p* = 0.01). Conversely, *CALR* mutation variants were similar in their hemoglobin levels (*p* = 0.8), leukocyte counts (*p* = 0.2) and major thromboses at or prior to ET diagnosis (*p* = 0.2). Notably, when considering arterial thrombosis, there was a trend of significance (*p* = 0.06) since more arterial events were documented in *CALR* type 1/1-like cases. Table 1 and Supplementary Table 1 list the clinical and laboratory features of the study population, stratified by driver mutational status.

**Table 1:** Presenting clinical and laboratory characteristics of 1000 patients with essential thrombocythemia (ET) stratified by driver mutation (*JAK2, CALR, MPL*, triple negative).

Variables	All patients *n* = 1000	*JAK2* mutated *n* = 659 (66%)	*CALR* mutated *n* = 187 (19%)	*MPL* mutated *n* = 44 (4%)	Triple negative *n* = 110 (11%)	*P*-value *JAK2 vs CALR*	*P*-value *JAK2 vs MPL*	*P*-value *JAK2 vs* Triple negative	*P*-value *CALR vs* Triple negative
Age in years, median (range)	59 (18–95)	62 (18–95)	53 (19–95)	59 (25–89)	53 (18–87)	**<0.001**	0.92	**<0.001**	0.51
Age ≥ 60 years, *n* (%)	484 (48)	353 (54)	69 (37)	21 (48)	41 (37)	<**0.001**	0.43	**0.002**	0.95
Female gender, *n* (%)	646 (65)	440 (67)	87 (46)	35 (80)	84 (76)	**<0.001**	0.08	**0.04**	**<0.001**
Hemoglobin g/dL, median (range)	14 (10–17.6)	14.2 (10–17.6)	13.8 (11–17.2)	13.1 (11.1–17)	13.7 (11.5–16.7)	**<0.001**	**<0.001**	**<0.001**	0.91
Leukocyte count, 10^9^/L, median (range)	8.5 (3.2–22)	8.7 (3.2–22)	8.0 (3.8–13.5)	7.7 (4–16.6)	8 (4.2–21.5)	**<0.001**	**0.004**	0.07	0.09
Leukocyte count > 11 × 10^9^/L, *n* (%)	160 (16)	120 (18)	15 (8)	7 (16)	18 (16)	<**0.001**	0.73	0.64	**0.03**
Platelet count, 10^9^/L, median (range)	715 (450–2088)	671 (450–1881)	826 (464–2088)	838 (451–1742)	725 (461–1700)	**<0.001**	**<0.001**	**0.005**	**0.002**
Platelet count ≥ 1000 × 10^9^/L, *n* (%)	156 (16)	70 (11)	52 (28)	11 (25)	23 (21)	<**0.001**	**0.004**	**0.002**	0.19
Platelet count ≥ 1500 × 10^9^/L, *n* (%)	96 (10)	22 (4)	67 (36)	4 (9)	3 (3)	<**0.001**	0.07	0.73	**0.001**
Cardiovascular risk factors, *n* (%)	518 (52)	364 (55)	84 (45)	23 (52)	47 (43)	**0.01**	0.71	**0.02**	0.71
Diabetes mellitus	62 (6)	45 (7)	7 (4)	2 (4)	8 (7)	0.13	0.55	0.86	0.18
Hypertension	347 (35)	244 (37)	57 (30)	17 (39)	29 (26)	0.09	0.83	**0.03**	0.45
Smoking	161 (16)	112 (17)	25 (13)	6 (14)	18 (16)	0.22	0.56	0.87	0.48
Hyperlipidemia	181 (18)	129 (20)	28 (15)	9 (20)	15 (14)	0.1	0.89	0.14	0.75
Palpable splenomegaly, *n* (%)	126 (13)	90 (14)	22 (12)	3 (7)	11 (10)	0.52	0.21	0.29	0.64
Abnormal karyotype, *n* (%)	30 (10)	22/197 (11)	3/62 (5)	1/15 (7)	4/25 (16)	0.10	0.63	0.48	0.08
*N* evaluable = 299									
Major thrombosis at or prior to diagnosis, *n* (%)	186 (19)	149 (23)	16 (9)	10 (23)	11 (10)	**<0.001**	0.94	**0.003**	0.68
Arterial thrombosis^a^	132 (13)	106 (16)	13 (7)	5 (11)	8 (7)	**0.001**	0.42	**0.02**	0.92
Venous thrombosis^b^	63 (6)	50 (8)	4 (2)	6 (14)	3 (3)	**0.007**	0.12	0.06	0.75
Major hemorrhage at or prior to diagnosis, *n* (%)	42 (4)	22 (3)	11 (6)	2 (4)	7 (6)	0.12	0.89	0.14	0.90
Microvascular symptoms, *n* (%)	293 (29)	176 (27)	60 (32)	23 (52)	34 (31)	0.17	**0.001**	0.36	0.83
IPSET^a^ thrombosis, *n* (%)									
Low	244 (24)	0(0)	136 (73)	24 (54)	84 (76)	**<0.001**	<**0.001**	<**0.001**	0.5
Intermediate	221 (22)	157 (24)	37 (20)	11 (25)	16 (15)				
High	535 (54)	502 (76)	14 (7)	9 (21)	10 (9)				
Revised IPSET-thrombosis, *n* (%)									
Very Low	200 (20)	0 (0)	114 (61)	20 (45)	66 (60)		**–**		0.81
Low	248 (25)	248 (38)	0 (0)	0 (0)	0 (0)				
Intermediate	104 (10)	0 (0)	57 (30)	14 (32)	33 (30)				
High	448 (45)	411 (62)	16 (9)	10 (23)	11 (10)				
IPSET-survival^b^, *n* (%)									
Low	380 (38)	202 (30)	105 (56)	17 (39)	56 (51)	<**0.001**	0.54	**<0.001**	0.53
Intermediate	437 (44)	314 (48)	65 (35)	18 (41)	40 (36)				
High	183 (18)	143 (22)	17 (9)	9 (20)	14 (13)				
Treatment instituted at diagnosis, *n* (%)									
Antiplatelet therapy	897 (90)	607 (92)	155 (83)	41 (93)	94 (85)	**0.001**	0.81	**0.02**	0.56
Cytoreductive therapy^c^	701 (70)	483 (73)	136 (72)	32 (73)	50 (45)	0.82	0.93	<**0.001**	**<0.001**
Systemic anticoagulation	111 (11)	75 (11)	20 (11)	8 (18)	8 (7)	0.84	0.18	0.20	0.33

Significant *p*-values are highlighted in bold.

^a^International prognostic score for thrombosis in ET (IPSET-thrombosis).

^b^International prognostic score for survival in ET (IPSET-survival).

^c^Cytoreductive therapies included hydroxyurea, anagrelide, interferon, busulphan, ruxolitinib.

### Epidemiology and risk factors of thrombosis and hemorrhage

At a median follow-up time of 8 years (range, 0.03–42.9), major thrombosis after diagnosis was documented in 143 (14%) patients, including 91 (9%) arterial and 65 (6%) venous events. Overall, the incidence of arterial events (1% patient-year) was higher than that of venous events (0.7% patient-year). Incidence rates of major arterial/venous thrombosis for *JAK2*, type 1/1-like *CALR*, type 2/2-like *CALR*, *MPL*-mutated and TN cases were 10%/8%, 10%/5%, 8%/4%, 14%/9% and 3%/2%, respectively.

To evaluate risk factors for arterial and venous thrombosis at diagnosis and during follow-up, we explored the impact of a number of variables. Concerning arterial thrombosis at or prior to diagnosis, univariate analysis reported as being statistically significant older age (*p* < 0.001), male sex (*p* = 0.01), a *JAK2*V617F mutated status (*p* < 0.001) and presence of at least one cardiovascular risk factor (*p* < 0.001); multivariate analysis confirmed the independent prognostic impact of older age (*p* < 0.001), *JAK2* mutation (*p* = 0.004) and ≥1cardiovascular risk factor (*p* < 0.001), but not male sex (*p* = 0.08). Conversely, neither platelet (*p* = 0.18) nor leukocyte counts (*p* = 0.77) were significant. Concerning venous thrombosis at or prior to diagnosis, the presence of *JAK2* mutation was the only significant risk factor identified (*p* = 0.02). No risk factor was identified as regarded the risk of hemorrhages at or prior to diagnosis.

Univariate analysis for arterial thrombosis-free survival (A-TFS) identified older age (*p* < 0.001), male sex (*p* = 0.03), presence of *JAK2* mutation (*p* = 0.04), cardiovascular risk factor (*p* = 0.002) and previous arterial event (*p* = 0.006) as risk factors for arterial thrombosis during follow-up. In contrast, antiplatelet treatment (*p* = 0.02; HR 0.3) and cytoreduction (*p* < 0.001; HR 0.4) had a protective effect on future arterial events. In age-adjusted univariate analysis, a borderline significance was retained for male sex (*p* = 0.05), previous arterial event (*p* = 0.06), antiplatelet treatment (*p* = 0.06), and the presence of *JAK2* mutation (*p* = 0.08). Multivariate analysis confirmed older age (for age ≥60 years; HR 2; *p* < 0.001) and the presence of *JAK2* mutation (HR 1.4; *p* = 0.02) as independent risk factors for future arterial thrombosis, whereas the protective effect of cytoreduction was confirmed (HR 0.4; *p* = 0.04).

Univariate analysis for venous thrombosis-free survival (V-TFS) identified the presence of *JAK2* mutation (*p* = 0.01) and previous venous event (*p* = 0.04) as predictors of a subsequent venous thrombosis; conversely, cytoreductive therapy displayed a protective role on venous thrombosis during follow-up (*p* = 0.006), whereas aspirin had a borderline significance (*p* = 0.09). Multivariate analysis for V-TFS identified *JAK2* mutation (HR 1.9; *p* = 0.03) as an independent predictor of cytoreductive therapy (HR 0.3; *p* = 0.01) as protective for future venous events.

Risk factors for major hemorrhage during follow-up by univariate analysis included older age (*p* < 0.001), higher leukocyte counts (*p* = 0.01), presence of cardiovascular risk factor(s) (*p* = 0.009), and cytoreductive therapy (*p* = 0.02). Multivariate analysis confirmed older age (HR 1.3; *p* = 0.01) and higher leukocyte count (for leukocyte count >11 × 10^9^/L, HR 2; *p* = 0.01). Results of univariate and multivariate analysis of risk factors for vascular events (arterial and venous thrombosis, and hemorrhage) at or after diagnosis of ET are summarized in Table 2.

**Table 2:**
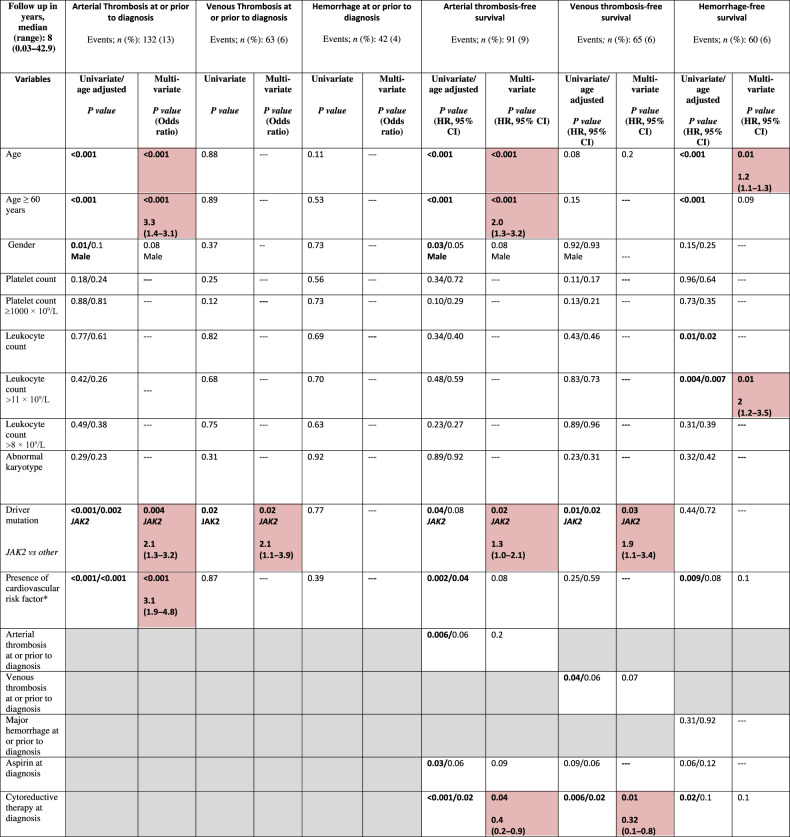
Univariate and multivariate analysis of associations/risk factors for vascular events (arterial/venous thrombosis and hemorrhage) at or after diagnosis among 1000 patients with essential thrombocythemia (ET), fully annotated for driver mutations.

Cardiovascular risk factor includes the presence of diabetes mellitus, hypertension, smoking, hyperlipidemia. Red shaded boxes represent variables of significance. Gray boxes represent variables that were not computed.

**Fig. 1:**
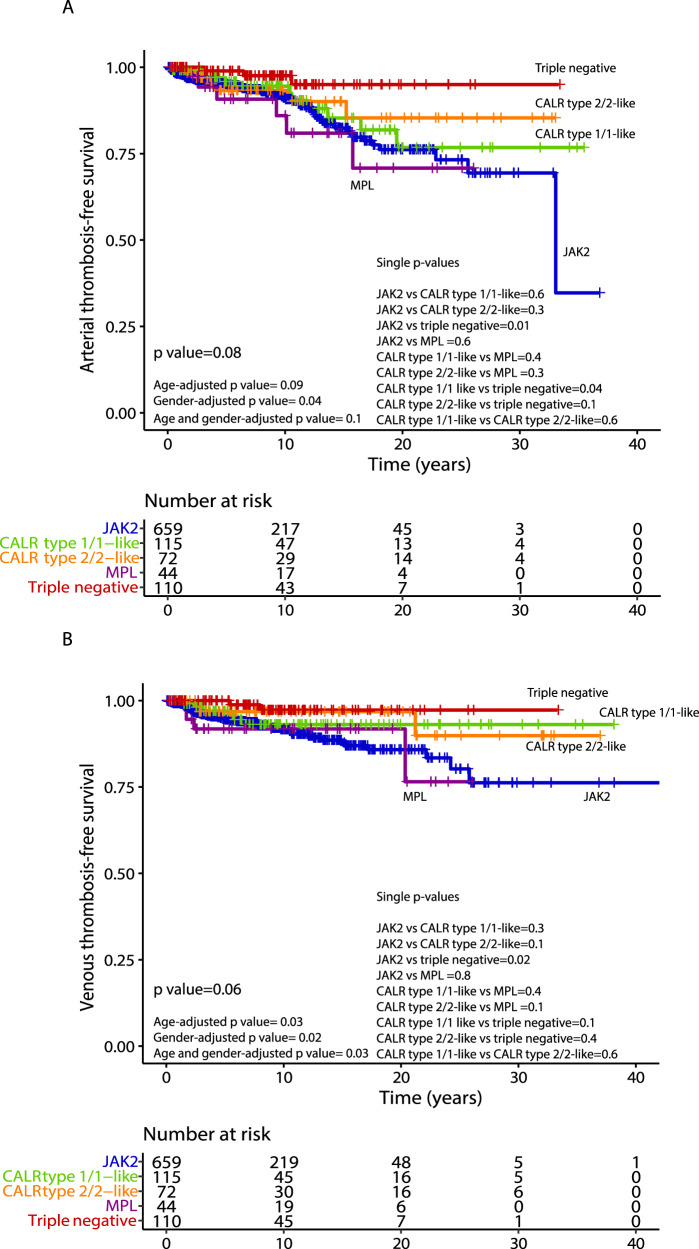
Arterial and venous thrombosis-free survival by driver mutation. Kaplan–Meier curves for arterial thrombosis-free survival (A-TFS, panel **A**) and venous thrombosis-free survival (V-TFS, panel **B**) of 1000 ET patients stratified by their driver mutation profile.

**Fig. 2:**
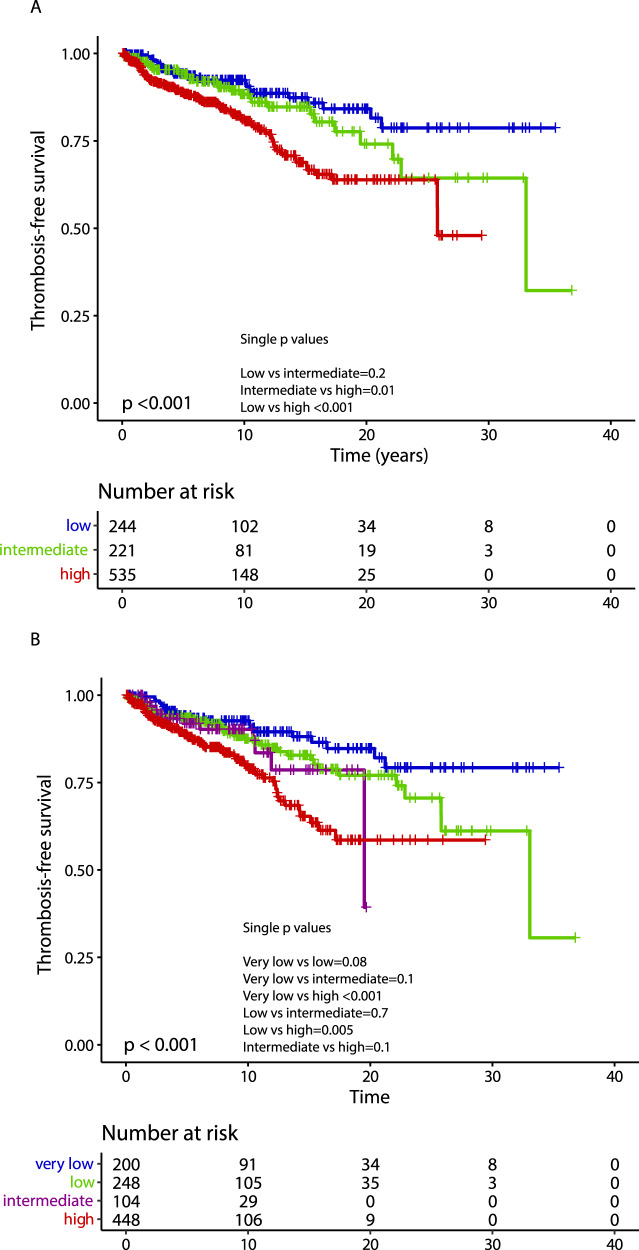
Thrombosis-free survival according to IPSET-thrombosis models. Kaplan–Meier curves of 1000 ET patients stratified in different risk categories according to the original International prognostic score in essential thrombocythemia (IPSET) for thrombosis (panel **A**) and the revised IPSET thrombosis (panel **B**) score.

After stratifying by driver mutations, A-TFS appeared to be better for TN vs *JAK2* patients (HR 4.1; *p* = 0.02), even when adjusted for age (HR 3.4; *p* = 0.04) and gender (HR 3.7; *p* = 0.03). Similar results were documented for *MPL* mutated patients vs TN (HR 5.4; *p* = 0.02), including after age (HR 5.1; *p* = 0.02) and gender adjustment (HR 5.6; *p* = 0.02). V-TFS was better for TN compared to *JAK2* mutated patients (HR 4.6; *p* = 0.03) also when age (HR 4.3; *p* = 0.04) and gender-adjusted (HR 4.9; *p* = 0.03). When gender-adjusted, a worst V-TFS for *MPL* mutated vs *CALR* type 1/1-like (HR 2.4; *p* = 0.02), *CALR* type 2/2-like (HR 6.4; *p* = 0.04) and TN (HR 7.1; *p* = 0.03) patients was documented. In Table 3, results of univariate analysis (age and gender adjusted) of vascular events in the follow-up, stratified by driver mutations, are reported. In Fig. 1A, B, A-TSF and V-TFS stratified by driver mutational status are shown, while Kaplan–Meier curves for the original and revised version of IPSET thrombosis are reported in Fig. 2A, B.

**Table 3:**
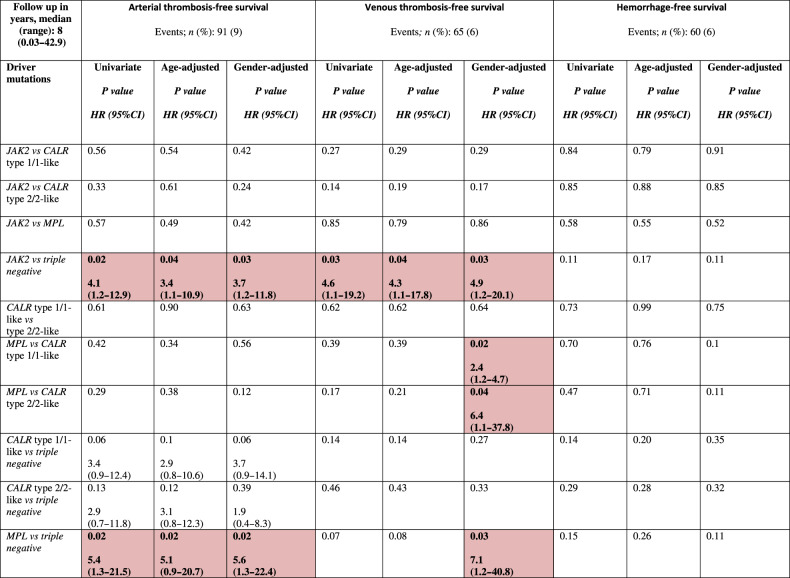
Univariate analysis of vascular events (arterial/venous thrombosis and hemorrhage) after diagnosis among 1000 patients with essential thrombocythemia (ET), fully annotated for driver mutations with *CALR* mutation type.

Red shaded boxes represent variables of significance.

### Overall survival

A total of 171 (17%) patients died during the follow-up period; the median follow-up time for all the 1000 patients and for living ones was 8 years (0.03–42.9) and 7.7 years (0.04–41.9), respectively. Causes of death were reported in 97 cases and included cardiovascular events (*n* = 16), disease progression to MF or blastic transformation (*n* = 21), infections (*n* = 9), major hemorrhage (*n* = 4), second solid malignancy (*n* = 15) and others (*n* = 32).

**Fig. 3:**
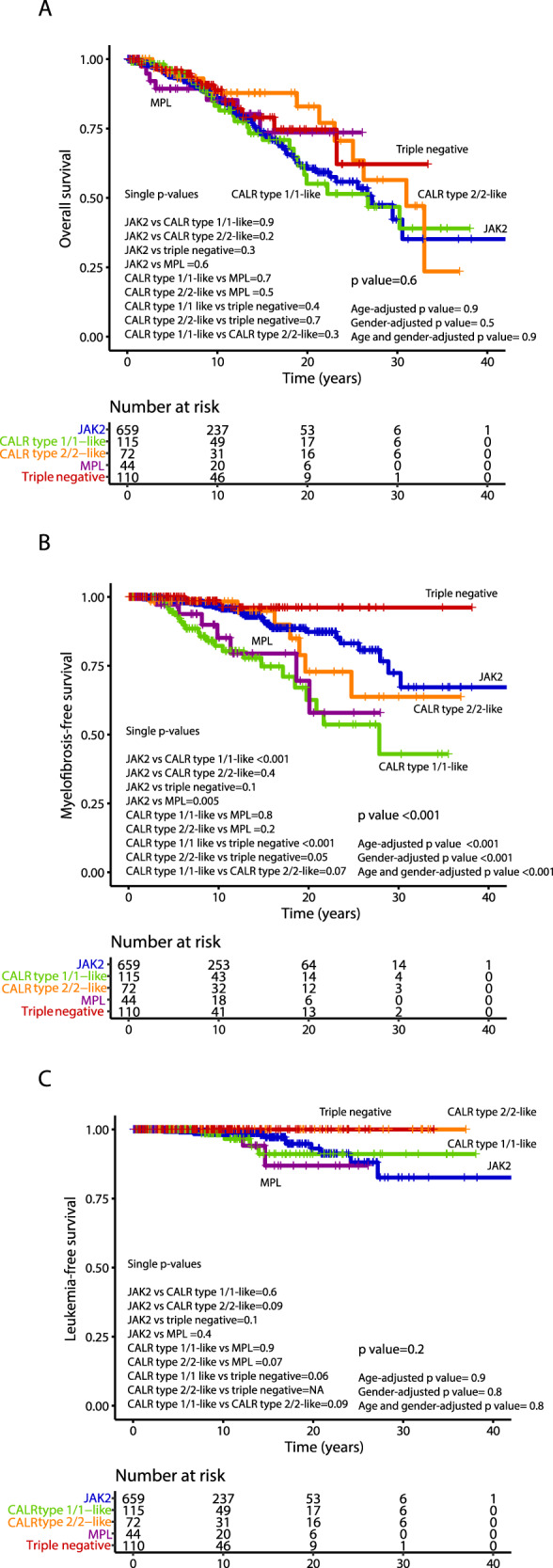
Overall, myelofibrosis-free and leukemia-free survival by driver mutation. Kaplan–Meier curves of 1000 ET patients for overall survival (panel **A**), myelofibrosis-free survival (panel **B**) and leukemia-free survival (panel **C**), stratified by their driver mutation profile.

**Fig. 4:**
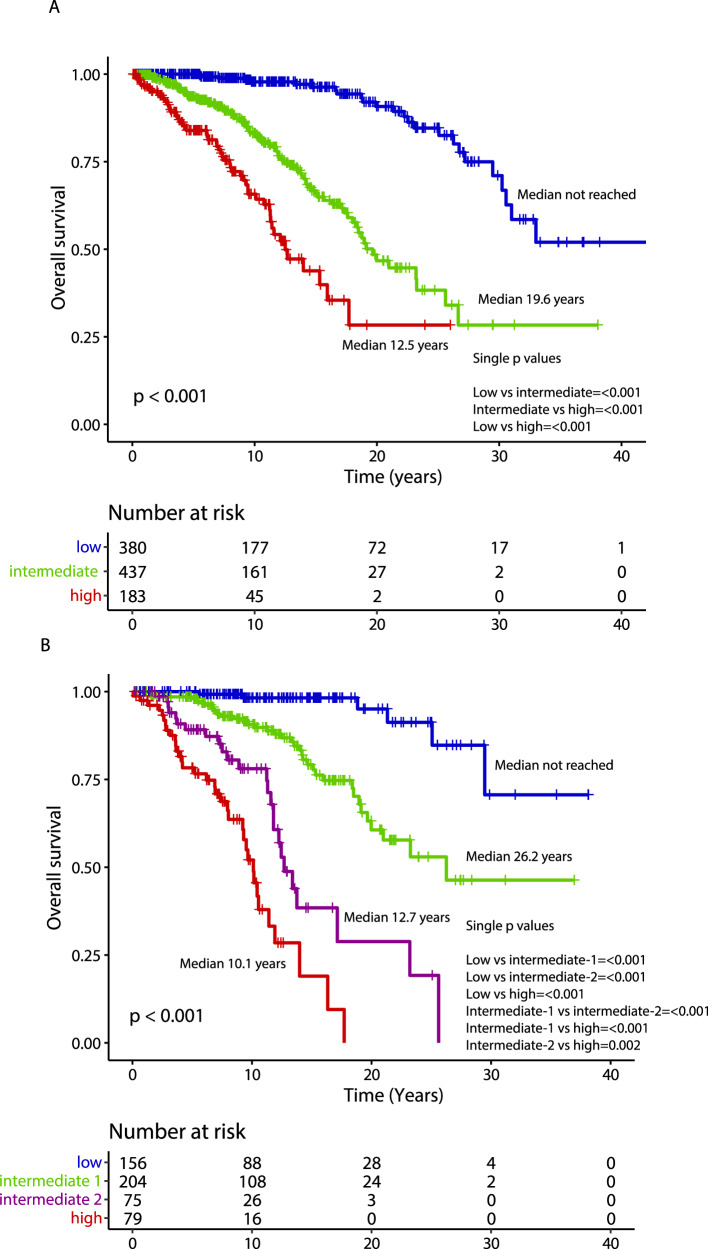
Overall survival according to IPSET and “triple AAA” models. Kaplan–Meier curves for overall survival of ET patients stratified in different risk categories according to the International prognostic score in essential thrombocythemia (IPSET) for survival (*n* = 1000; panel **A**) and the “triple AAA” model (Age, Absolute neutrophil count, Absolute Lymphocyte count, *n* = 514; panel **B**).

Median overall survival was 27.1 years, with 10-year, 20-year and 30-year survival rates of 86%, 64% and 43%, respectively. The following variables were flagged by univariate analysis as being significantly associated with OS: older age (*p* < 0.001), male gender (*p* = 0.001), higher leukocyte counts (*p* < 0.001), presence of cardiovascular risk factor(s) (*p* = 0.005), arterial thrombosis at or before ET diagnosis (*p* = 0.005), ANC ≥ 8 × 10^9^/L (*p* = 0.003) and ALC < 1.7 × 10^9^/L (*p* = 0.002); no significant association with any driver mutation was observed. Multivariate analysis identified older age (*p* < 0.001), male gender (HR 1.9; *p* < 0.001), ANC ≥ 8 × 10^9^/L (HR 1.8; *p* = 0.01) and ALC < 1.7 × 10^9^/L (HR 1.2; *p* = 0.03) as independent predictors of survival, unlike for cardiovascular risk factors (*p* = 0.36) and arterial thrombosis at or before ET diagnosis (*p* = 0.21). The overall survival Kaplan–Meier curve for patients stratified by driver mutational status is depicted in Fig. 3A, whereas survival curves analysis based on IPSET survival and “triple AAA” models are shown in Fig. 4A, B. Univariate and multivariate analysis of risk factors for overall survival are reported in Table 4.

**Table 4:**
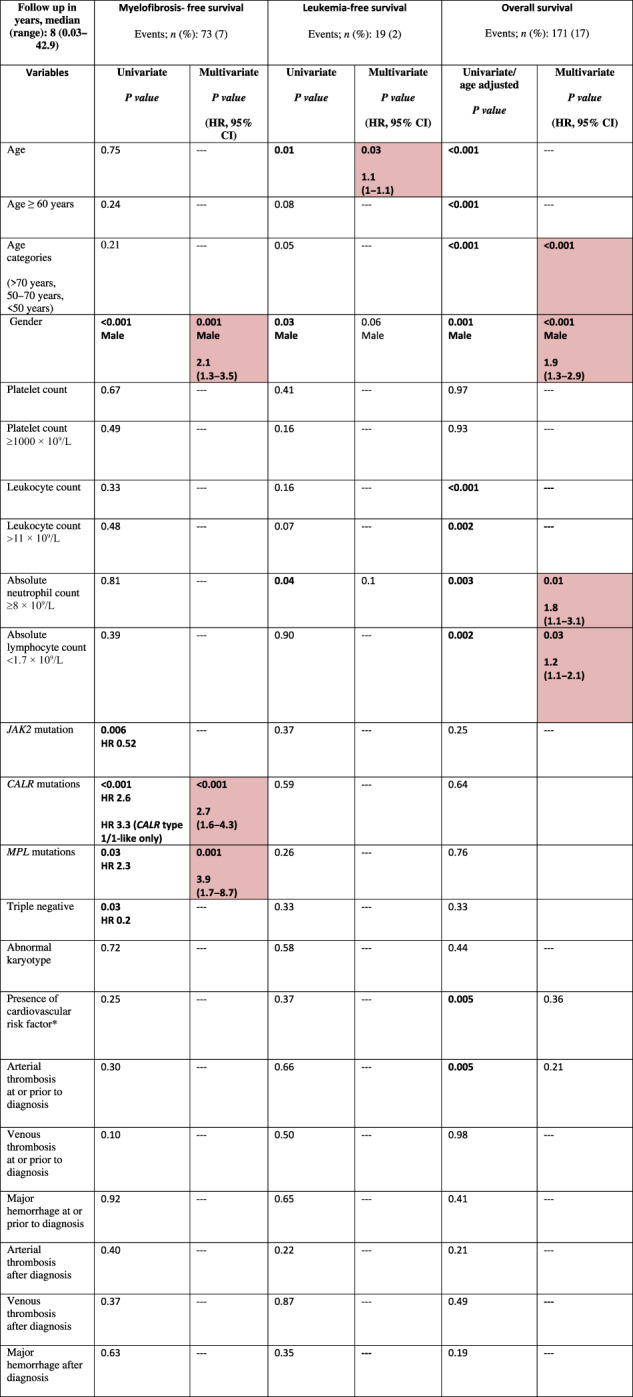
Univariate and multivariate analysis of risk factors for disease transformation (myelofibrosis and blast phase) and overall survival among 1000 patients with essential thrombocythemia (ET), fully annotated for driver mutations.

Shaded boxes represent variables of significance.

### Progression to post-ET myelofibrosis

Transformation to MF was experienced by 73 patients (7%), accounting for 10-year and 20-year incidence rates of 6% and 20%, respectively. The overall incidence of fibrotic transformation was 10% for *JAK2* mutated, 20% for type 1/1-like, 10% type 2/2-like *CALR*-mutated, 16% *MPL*-mutated and 2% for TN cases. The difference was statistically significant for *MPL* vs *JAK2* (HR 1.8, 95% CI 1.2–2.7; *p* = 0.005), *CALR* type 1/1-like vs *JAK2* (HR 3.2, 95% CI 1.8–5.6; *p* < 0.001), and *CALR* type 1/1-like vs TN (HR 9.6, 95% CI 2.2–41.4; *p* < 0.001). Moreover, *CALR* type 1/1-like patients were more prone to have fibrotic progression vs *CALR* type 2/2-like (HR 2.1; 95% CI 0.9–5.1; *p* = 0.07).

Univariate analysis for MFS highlighted the prognostic impact of *CALR* (*p* < 0.001) and *MPL* mutation (*p* = 0.03), along with male gender (*p* < 0.001). All these variables *CALR* (HR 2.7; *p* < 0.001), *MPL* (HR 3.9; *p* = 0.001) and male gender (HR 2.1; *p* = 0.001) were confirmed as independent predictors of fibrotic progression in multivariate analysis. The MFS curves resulting from stratifying patients by driver mutational status are reported in Fig. 3B. Univariate and multivariate analyses of risk factors for MF transformation are reported in Table 4.

### Transformation to blast phase

At the time of the last follow-up, blastic transformation was documented in 19 cases (2%). The overall incidence of blastic transformation was 2% for *JAK2* mutated, 3% for type 1/1-like and 4% for *MPL*-mutated. Of interest, no blastic progression was documented in *CALR* type 2/2-like and TN cases. Univariate analysis for LFS identified older age (*p* = 0.01), male gender (*p* = 0.03) and ANC ≥ 8 × 10^9^/L (*p* = 0.04), while only the former was confirmed to be significant in multivariate analysis (HR 1.1; *p* = 0.03). The LFS curves obtained by stratifying patients according to their driver mutational status are reported in Fig. 3C. Univariate and multivariate analyses of risk factors for blast phase transformation are reported in Table 4.

## Discussion

The current retrospective study performed in a large, monocentric reference center for diagnosis and management of MPN in Florence, Italy, constitutes, to the best of our knowledge, the largest single-center series of ET patients fully annotated for driver mutations ever reported. Thanks to the information accumulated in the database spanning 40 years, we were able to provide extensive information on disease characteristics, course and evolution and perform an in-depth analysis of prognostic factors for overall, leukemia-free, myelofibrosis-free, arterial and venous thrombosis-free survival, and validate contemporary prognostic models for thrombosis and survival. Presenting clinical and laboratory characteristics of our cohort are in line with previously described differences in smaller series among driver mutation categories [25–27]. *CALR* mutated and TN patients were younger compared to *JAK2* and *MPL* mutated cases. Concerning blood counts at diagnosis, median hemoglobin and leukocyte values were higher in *JAK2* mutated than in the other genotypes, while higher platelet counts were reported in *CALR* (type 2/2-like more than type 1/1-like) and *MPL* cases. At the time of diagnosis, major thromboses were documented in 19%, with a predominance of arterial (13%) versus venous (6%) events. Major hemorrhages were reported in 4% of patients. Of note, the incidence of both arterial and venous thrombosis was lower in *CALR* mutated and TN patients, compared to *JAK2* and *MPL* mutated.

One major value of the current study is the long-term follow-up data that, although with intrinsic limitations of a retrospective series, enabled estimation of thrombosis, survival, and transformation rates in a contemporary series of annotated cases. Concerning thrombosis, we confirmed the higher rate of arterial and venous events experienced after diagnosis by *JAK2* mutated patients when compared with all the others, especially with TN patients; this detrimental effect remained significant in multivariate analysis including age, gender, thrombosis history, presence of cardiovascular risk factors and also cytoreductive and antiplatelet therapy. Of note, *MPL* mutation pointed to a significantly higher risk of arterial thrombotic event vs TN, also when age and gender-adjusted, and venous thrombotic events versus either *CALR* type 1/1-like and type 2/2-like patients (when gender adjusted). Moreover, a higher risk of arterial events for *CALR* type 1/1-like mutated vs TN patients was observed, although it was no longer significant when age, gender, and treatments were introduced in multivariate analysis. An important, clinically relevant finding was the confirmation that TN patients have a very low rate of thrombosis. Furthermore, an independent protective effect of aspirin, especially for arterial events, and cytoreductive therapy for both arterial and venous events was observed. In the present study, we stratified patients at diagnosis according to either the original or revised version of the IPSET thrombosis score. Overall, a clear-cut significant difference between low/very low and high-risk categories was documented in both models, whereas no significant differences were highlighted in low/very-low vs intermediate risk, as recently reported in a large multicentric study involving 1366 patients from the Spanish registry [28].

Considering the whole study population, the median overall survival was 27.1 years; in total, 7% of the patients progressed to PET-MF, and the 10-year and 20-year incidence rate was 6% and 20%, respectively. Driver mutational status did not impact overall and leukemia-free survival; conversely, *CALR* (type 1/1-like more than type 2/2-like) and *MPL* mutations were significantly associated with a higher risk of MF progression, as previously reported [19, 25, 29–32].

Multivariate analysis for OS identified independent impact of older age, male gender, ANC ≥ 8 × 10^9^/L and ALC < 1.7 × 10^9^/L; these results were in line with previous reports [21, 33]. The application of the recent, four-tiered, “triple AAA” survival model to the current patient cohort (including 514 evaluable patients) resulted in median survival estimated not reached for low, 26.2 years for intermediate-1, 12.7 years for intermediate-2 and 10.1 for high risk. In comparison, the use of the three-tiered IPSET survival model applied to the whole patients’ cohort, resulted in median survival not reached for low, 19.6 years for intermediate and 12.5 years for the high-risk category. As recently reported, the “triple AAA” model performed better over IPSET survival also in this series [21].

We acknowledge potential limitations in the interpretation of findings reported herein, mainly concerning the retrospective nature of the study that may harbor intrinsic selection biases.

However, findings concerning thrombosis are relevant for patient management, including the observation that cytoreductive treatment and aspirin, to a lesser extent, seemed to mitigate future thrombotic events, as well as reinforcing the informativeness of available prognostic models for thrombosis. On the contrary, data on overall survival and fibrotic progression may be more useful for patient counseling and disease monitoring rather than for shaping treatment since no drug has ever been shown convincingly to exert disease-modifying activity in ET.

While prospective multicenter studies are preferred to retrospective ones, they are definitely difficult to be conducted in disease like ET, where survival exceeds several decades; therefore, the hope is that information such as those presented in this real-world, extensively annotated, series of patients can represent a valid source for interpreting current literature and planning future, prospective studies.

### Supplementary information

Supplemental Table 1

## Data Availability

The data that support the findings of this study are available from the corresponding author upon request. The data are not publicly available due to privacy or ethical restrictions.
