# Supplementary material for: One thousand patients with essential thrombocythemia: the Florence-CRIMM experience
Source: Blood Cancer J. 2024 Jan 18;14(1):10. doi: 10.1038/s41408-023-00968-7 (PMC10796728; doi:10.1038/s41408-023-00968-7)
Supplement: Supplementary file 1 — Supplemental Table 1 [file 41408_2023_968_MOESM1_ESM.docx]

**Supplemental Table 1. Presenting clinical and laboratory characteristics of 846 patients with essential thrombocythemia (ET) stratified by *JAK2 vs* Type 1/1-like vs Type 2/2-like *CALR* mutations.**

| **Variables** | **All patients**  ***n*=838** | ***JAK2* mutated**  ***n* =659 (78%)** | ***Type 1/1-like CALR* mutated**  ***n*=115 (13%)** | ***Type 2/2-like CALR* mutated**  ***n*=72 (9%)** | ***P-value***  ***JAK2 vs Type 1 CALR*** | ***P-value***  ***JAK2 vs Type 2 CALR*** | ***P-value***  ***Type 1 vs Type 2 CALR*** |
| --- | --- | --- | --- | --- | --- | --- | --- |
| Age in years, median (range)   - Age ≥ 60 years, n (%) | 60 (18-95)  418 (50) | 62 (18-95)  353 (54) | 55 (21-85)  47 (41) | 50 (20-95)  22 (31) | **0.03**  **0.01** | **<0.001**  **<0.001** | 0.07  0.2 |
| Female gender, n (%) | 523 (62) | 440 (67) | 48 (42) | 39 (54) | **<0.001** | **0.03** | 0.09 |
| Hemoglobin g/dL, median (range) | 14.1 (10-17.6) | 14.2 (10-17.6) | 13.8 (11-17.2) | 13.4 (11-16) | **<0.001** | **<0.001** | 0.8 |
| Leukocyte count, 10^9^/L, median (range)  - Leukocyte count > 11 x 10^9^/L, n (%) | 8.5 (3.2-22)  135 (16) | 8.7 (3.2-22)  120 (18) | 8.2 (3.8-13.2)  11 (10) | 7.5 (3.8-13.5)  4 (6) | **0.002**  **0.03** | **<0.001**  **0.007** | 0.2  0.3 |
| Platelet count, 10^9^/l, median (range)  - Platelet count ≥ 1000 x 10^9^/L, n (%)  - Platelet count ≥ 1500 x 10^9^/L, n (%) | 698 (450-2088)  120 (14)  88 (10) | 671 (450-1881)  70 (11)  22 (4) | 780 (464-2000)  27 (23)  43 (37) | 923 (548-2088)  25 (35)  24 (33) | **<0.001**  **<0.001**  **<0.001** | **<0.001**  **<0.001**  **<0.001** | **0.01**  **0.04**  0.1 |
| Cardiovascular risk factors, n (%)  -Diabetes mellitus  -Hypertension  -Smoking  -Hyperlipidemia | 446 (53)  52 (6)  299 (36)  137 (16)  157 (19) | 364 (55)  45 (7)  244 (37)  112 (17)  129 (20) | 50 (47)  6 (6)  33 (31)  13 (12)  17 (16) | 32 (44)  1 (1)  22 (31)  12 (17)  11 (15) | 0.1  0.6  0.2  0.2  0.4 | 0.08  0.07  0.3  0.9  0.4 | 0.8  0.1  0.9  0.4  0.9 |
| Palpable splenomegaly, n (%) | 111 (13) | 90 (14) | 13 (11) | 9 (12) | 0.5 | 0.8 | 0.7 |
| Abnormal karyotype, *n* (%)  N evaluable=258 | 25 (10) | 22 (11) | 1 (2) | 2 (9) | 0.09 | 0.82 | 0.2 |
| Major thrombosis at or prior to diagnosis, *n* (%)   - Arterial thrombosis^*^ - Venous thrombosis^#^ | 165 (20)  119 (14)  54 (6) | 149 (23)  106 (16)  50 (8) | 12 (10)  11 (10)  2 (2) | 4 (5)  2 (3)  2 (3) | **0.007**  **0.04**  **0.02** | **<0.001**  **0.003**  0.09 | 0.2  0.06  0.7 |
| Major hemorrhage at or prior to diagnosis, n (%) | 33 (4) | 22 (3) | 7 (6) | 4 (6) | 0.2 | 0.9 | 0.9 |
| Microvascular symptoms, n (%) | 234 (28) | 176 (27) | 37 (32) | 23 (32) | 0.2 | 0.3 | 0.9 |
| Revised IPSET-thrombosis^*^, n (%)   - Very Low - Low - Intermediate - High | 110 (13)  248 (30)  53 (6)  427 (51) | 0 (0)  248 (38)  0 (0)  411 (62) | 67 (58)  0 (0)  36 (31)  12 (11) | 47 (65)  0 (0)  21 (29)  4 (5) | - | - | 0.4 |
| IPSET-survival^#^, n (%)   - Low - Intermediate - High | 303 (36)  375 (45)  160 (19) | 202 (30)  314 (48)  143 (22) | 59 (51)  43 (38)  13 (11) | 46 (64)  22 (31)  4 (5) | **<0.001** | **<0.001** | 0.2 |
| Treatment instituted at diagnosis, n (%)   - Aspirin - Cytoreductive therapy^$^ - Systemic anticoagulation | 754 (90)  613 (73)  94 (11) | 607 (92)  483 (73)  75 (11) | 100 (87)  82 (71)  16 (14) | 55 (76)  54 (75)  4 (5) | **0.04**  0.6  0.4 | **<0.001**  0.7  0.1 | 0.1  0.6  0.07 |

*Revised International prognostic score for thrombosis in ET (Revised IPSET-thrombosis).

# International prognostic score for survival in ET (IPSET-survival).

$ cytoreductive therapies included hydroxyurea, anagrelide, interferon, busulphan, ruxolitinib

Significant p values are highlighted in bold.
